# The values of neutrophil-lymphocyte ratio and/or prostate-specific antigen in discriminating real Gleason score ≥ 7 prostate cancer from group of biopsy-based Gleason score ≤ 6

**DOI:** 10.1186/s12885-017-3614-9

**Published:** 2017-09-06

**Authors:** Hanfeng Wang, Liangyou Gu, Yongjie Wu, Dan Feng, Junyao Duan, Xiaocong Wang, Yong Huang, Shengpan Wu, Jianwen Chen, Guangda Luo, Xu Zhang

**Affiliations:** 1 Department of Urology, Chinese PLA General Hospital/PLA Medical School, Beijing, 100853 People’s Republic of China; 2 Department of General Surgery, Chinese PLA 264 Hospital, Taiyuan, 030000 China; 3 Hospital Management Institute, Medical Statistic Division, Chinese PLA General Hospital/PLA Medical School, Beijing, 100853 China; 4 Department of Pathology, Chinese PLA General Hospital/PLA Medical School, Beijing, 100853 China

**Keywords:** Prostate cancer, Neoplasm grading, Systemic inflammatory index, Watchful waiting

## Abstract

**Background:**

The discrepant concordance between biopsy and radical prostatectomy (RP) specimen are well reported. To validate the clinical usefulness of neutrophil-lymphocyte ratio (NLR) in discriminating real GS ≥ 7 PCa from biopsy-based GS ≤ 6 PCa in comparison with serum total prostate-specific antigen (tPSA) and value of their combination.

**Methods:**

One hundred one patients who underwent physical examinations incidentally found elevated tPSA and subsequently received biopsy with a conclusion of GS ≤ 6 and RP with an interval of 4-6 weeks after biopsy were enrolled. NLR and tPSA were obtained within 15 days prior to biopsy. Logistic regression model was applied appropriately; McNemar tests and AUC model were performed to evaluate differences among tPSA, NLR and their combination and corresponding diagnostic power respectively.

**Results:**

The pathological results from RP specimen comprised 61 patients with GS ≤ 6 and 100 patients with GS ≥ 7. Higher tPSA and NLR were significantly associated with patients with actual GS ≥ 7 (All *P* < 0.05) concurrently. Multivariate logistic regression indicated that tPSA (OR = 1.088, 95% C.I. = 1.029-1.151, *P* = 0.003) and NLR (OR = 1.807, 95% C.I. = 1.021-3.200, *P* = 0.042) could be independent predictors for GS groupings. Under cutoff value of 14.09 ng/ml for tPSA and 2.25 for NLR, the sensitivity, specificity and accuracy were 60.0%, 80.3% and 67.7% for tPSA, 42%, 88.5% and 59.6% for NLR, and 71.0%, 75.4% and 72.7% for combination of tPSA and NLR (tPSA + NLR) respectively. The sensitivity of tPSA + NLR was significantly higher in comparison with tPSA (*P* = 0.001) and NLR (*P* < 0.001). Except for sensitivity, no significant difference was found between tPSA and NLR in specificity (*P* = 0.227) and accuracy (*P* = 0.132). tPSA got the largest AUC with 0.732 (*p* < 0.001, 95% C.I.: 0.651-0.813).

**Conclusions:**

Serum tPSA and NLR were significantly elevated among GS ≥ 7 PCa concurrently. The combination of tPSA and NLR might have additional benefit to biopsy on discriminating real GS ≥ 7 Pca from biopsy-based GS ≤ 6 PCa. More stratification models and prospectively multicenter studies are necessary.

## Background

Prostate cancer (PCa) has become the most common malignancy among men in several developed countries and its incidence has ranked 7 In China [1–3]. As its cancer-specific mortality is relatively low and almost 80% of men diagnosed with PCa ultimately died of other causes [4], the treatment for PCa still remains controversial, particular for Gleason score (GS) ≤6. In recent years, active surveillance (AS) has been a universally accepted approach for men with low risk localized PCa. Under using D’Amico criteria, a large proportion of patients with GS ≤ 6 PCa have no need to undergo radical treatment immediately [5]. However, the GS given by biopsy were occasionally underestimated or overestimated in comparison with the pathology of radical prostatectomy (RP) specimens. A study including 1363 patients conducted by Rajinikanth A et al. [6] revealed that biopsy and radical prostatectomy GS categories correlated exactly in 937 (69%) men with underestimation in 361 (26%) and overestimation in 65 (5%). In our clinical work, the situation above happened occasionally as well, which would lead inappropriate management for patients with different GS levels, such as overtreatment for actual GS ≤ 6 PCa or delay timely intervention for actual GS ≥ 7 PCa.

According to Lima NG et al. [7], the patients with preoperative PSA levels < 10 ng/ml had higher concordance of Gleason 3 + 3 scores between biopsy and pathology of RP than those with preoperative PSA levels ≥ 10 ng/ml (*P* = 0.023). Simultaneously, neutrophil-lymphocyte ratio (NLR), a cancer-related systemic inflammatory marker, may predict PCa in men undergoing prostate needle biopsy with or without combination with F/T PSA ratio and was found to differ with regard to histology of prostate biopsy [8, 9]. Besides, studies conducted by Gokce MI [10] also showed that NLR could be a predictor of GS upgrading in patients with low-risk PCa. However, there are few studies concerning the clinical usefulness of NLR in improving the accuracy rate and diagnostic power of biopsy and discriminating real GS ≥ 7 Pca from biopsy-based GS ≤ 6 PCa in comparison with serum total PSA (tPSA).

The aim of our study was to evaluate whether tPSA has diagnostic differences with NLR and assess their clinical usefulness in detecting real GS ≥ 7 PCa among those biopsy-based GS ≤ 6 PCa.

## Methods

Men diagnosed with PCa from January of 2014 to May of 2016 were selected from database of single-institution and this retrospective study was approved by institutional review board of PLA General Hospital. The following selection criteria were applied: 1. elevated tPSA was incidentally found from physical examinations; 2. all pathological results of biopsy specimens were less or equal to 6; 3. the patient had no diagnosed acute or chronic inflammation in any system and no history of taking antibiotics within the latest 2 weeks; 4. the patient had no hematological and immune diseases; 5. the patient had not received neoadjuvant hormonal therapy (NHT) after biopsy and before radical surgery; 6. the patient was prostate cancer-specific; 7. the patient had no 5-alpha-reductase inhibitors taken history. One hundred ninety-eight 198 patients were identified based on criteria above. However, 21 patients without pre-biopsy regular blood test, 16 patients with missing serum PSA data, who were all excluded from this study. Therefore, 161 patients were enrolled in this study.

### Data collection

Baseline characteristics (age, body mass index, tPSA, neutrophil count, lymphocyte count, and NLR) and lifestyle behaviors (alcohol and smoking) were collected through the database and medical records. All blood specimens were collected and tested by a single laboratory within 15 days before 12-core transperineal ultrasound-guided prostate biopsy. Serum total PSA levels (ng/ml), neutrophil count (10^9 cells/L) and lymphocyte count (10^9 cells/L) were acquired from blood tests. NLR was calculated as neutrophil count divided by lymphocyte count.

### Histopathological evaluation

The pathological characteristics of specimens from 12-core prostate biopsy and RP (prostate volume, Gleason score, pathologic T stage and N stage) were studied respectively and reviewed separately by two genitourinary pathologists who were blinded to original histopathological results and blood test outcomes.

### Statistical analyses

Patients were categorized into groups of GS ≤ 6 and GS ≥ 7 basing on pathology of RP specimens. Baseline characteristics were compared between two groups using Student’s t test or nonparametric test for continuous variables and chi-square test for categorical variables respectively. Univariate and multivariate logistic regression were performed to identify predictive variables for GS groupings. Correlate analysis was used appropriate. ROC curve were performed to calculate the cutoff values. In order to increase the sensitivity of detecting GS ≥ 7 PCa from biopsy-based GS ≤ 6 PCa, we defined combination of tPS and NLR (tPSA + NLR) was positive when either tPSA or NLR indicated GS ≥ 7 under cutoff values. Differences in sensitivity, specificity, positive predictive value (PPV), negative predictive value (NPV) and accuracy were compared by using McNemar tests. AUC model analyses were used to evaluate their diagnostic power respectively. All statistical analyses were conducted in Statistical Package for Social Sciences (SPSS) version 22.0 (SPSS Inc., Chicago, USA). Two-sided *P* value of <0.05 was considered statistically significant.

## Results

Of the 161 patients, the outcomes of reviews conducted by two separated pathologists were consistent. On the basis of RP pathological review, there were 61 (37.9%) patients had histological GS ≤ 6 (3 patient with GS = 4, 5 patients with GS = 5 and 53 patients with GS = 6) and 100 (62.1%) had GS ≥ 7 (85 patients with GS = 7, 10 patients with GS = 8 and 5 patients with GS = 9) respectively. All the patients underwent radical prostatectomy. In comparison with a median of 10.00 ng/ml for tPSA and a median of 1.68 for NLR in group of GS ≤ 6, the patients with GS ≥ 7 had significant higher tPSA and NLR with median of 15.10 (*P* < 0.001) and 1.96 (*P* = 0.008) respectively. However, the following variables had no statistical significance between two groups: age (*P* = 0.541), prostate volume (*P* = 0.989), neutrophil count (*P* = 0.201), lymphocyte count (*P* = 0.370), body mass index (BMI) (*P* = 0.631), smoking history (*P* = 0.086), alcohol history (*P* = 0.258), positive core (*P* = 0.570), max cancer of core (*P* = 0.489), pT stage (*P* = 0.194), pN stage (*P* = 0.332) (Table 1).

**Table 1:** Clinicalpathologic features between study groups

Variables	GS ≤ 6	GS ≥ 7	*P* Value
	*N* = 61	*N* = 100	
Age (IQR), years	65(57, 72)	65(61, 70)	0.514
tPSA (IQR), ng/ml	10.00(6.81, 13.54)	15.10(9.81, 20.70)	<0.001
Prostate volume (IQR),ml	38.69(35.41, 42.44)	38.31(34.81, 43.54)	0.989
Neutrophil count (IQR), 10^9^/L	3.30(2.89, 4.00)	3.59(2.86, 4.74)	0.201
Lymphocyte count (IQR), 10^9^/L	1.96(1.57, 2.34)	2.06(1.65, 2.47)	0.370
NLR (IQR), %	1.68(1.32, 2.07)	1.96(1.43, 2.49)	0.008
BMI (Mean ± SD), kg/m^2^	25.66 ± 2.57	25.19 ± 2.96	0.631
Smoking History, No.(%)			
No	48(78.9%)	66(66.0%)	0.086
Yes	13(21.3%)	34(34.0%)	
Alcohol History, No.(%)			
No	42(68.3%)	60(60.0%)	0.258
Yes	19(31.7%)	40(40.0%)	
Positive cores(IQR), No.	6(5, 8)	6(4, 8)	0.570
Max cancer of core(IQR), %	50(30, 65)	50(30, 60)	0.489
Pathologic T Stage, No.(%)			
pT2	50(82.0%)	73(73.0%)	0.194
pT3	11(18.0%)	27(27.0%)	
Pathologic N Stage, No.(%)			
pN0	53(86.9%)	81(81.0%)	0.332
pN1	8(13.1%)	19(19.0%)	

Groups of GS ≤ 6 and GS ≥ 7 PCa were divided basing on pathology of radical prostatectomy specimens

In univariate logistic regression analysis, tPSA (OR = 1.098; 95% C.I. = 1.036-1.164; *P* = 0.002) and NLR (OR = 1.833; 95% C.I. = 1.004-3.347; *P* = 0.049) were statistically significant between two groups. Both tPSA and NLR included, multivariate logistic regression model demonstrated that tPSA (OR = 1.088; 95% C.I. = 1.029-1.151; *P* = 0.003) and NLR (OR = 1.807; 95% C.I. = 1.021-3.200; *P* = 0.042) still remained significantly associated with GS groupings. In other words, higher pretreatment values of tPSA and NLR predicted higher GSs. (Table 2).

**Table 2:** Univariate and Multivariate logistic regression for analyzing candidate predictors of GS groupings

Variables	Univariate			Multivariate		
	*p*	OR	95% C.I.for EXP(B)	*p*	OR	95% C.I.for EXP(B)
Age	0.828	1.006	0.955-1.059	-		
tPSA	0.002	1.098	1.036-1.164	0.003	1.088	1.029-1.151
NLR	0.049	1.833	1.004-3.347	0.042	1.807	1.021-3.200
BMI	0.910	0.993	0.873-1.129	-		
Prostate volume	0.606	0.984	0.926-1.046	-		
Smoking History	0.690	1.191	0.504-2.814	-		
Alcohol History	0.204	1.719	0.746-3.96	-		

**Fig. 1:**
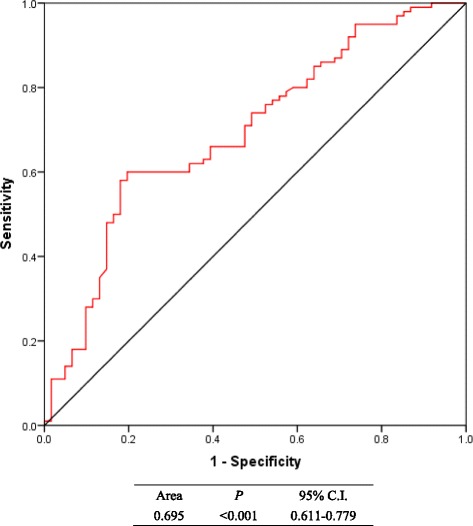
ROC curve for calculating cutoff value of tPSA (with corresponding sensitivity of 60.0% and specificity of 80.3%

**Fig. 2:**
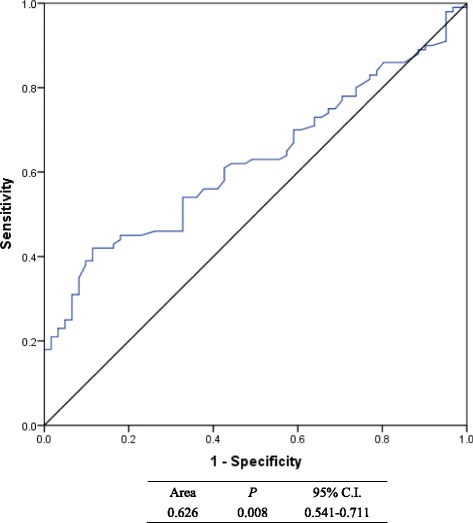
ROC curve for calculating cutoff value of NLR (with corresponding sensitivity of 42.0% and specificity of 88.5%)

By applying ROC curve method, the cutoff values of tPSA and NLR were 14.09 ng/ml with AUC = 0.695 (95% C.I. = 0.611-0.779) and 2.25 with AUC = 0.626 (95% C.I. = 0.541-0.711) respectively (Figs. 1 and 2). Depending on the cutoff values, we classified patients and assessed clinical usefulness of tPSA, NLR and tPSA + NLR in discriminating real GS ≥ 7 Pca from those biopsy-based GS ≤ 6 PCa. The results revealed that tPSA got a accuracy rate of 67.7% (109/161) with a sensitivity of 60.0% (60/100) and a specificity of 80.3% (49/61); NLR got a accuracy rate of 59.6% (96/161) with a sensitivity of 42% (42/100) and a specificity of 88.5% (54/61) and tPSA + NLR got a accuracy rate of 72.7% (117/161) with a sensitivity of 71.0% (71/100) and a specificity of 75.4% (46/61) respectively. Accordingly, except for between sensitivities (*P* = 0.006), no statistical difference was found between tPSA and NLR in specificity (*P* = 0.227) and in accuracy (*P* = 0.132). Although tPSA + NLR got the highest accuracy rate with 67.7%, it had no statistical significance in comparison with tPSA (*P* = 0.330). However, this combination also got the highest sensitivity in detecting real GS ≥ 7 Pca from biopsy-based GS ≤ 6 PCa, which had statistical significance in comparison with that of tPSA (*P* = 0.001) and that of NLR (*P <* 0.001) (Table 3).

**Table 3:** Diagnostic performances of three methods (tPSA, NLR and tPSA + NLR)

	tPSA	NLR	*P* value	tPSA + NLR	tPSA	*P* value	tPSA + NLR	NLR	*P* value
Sensitivity	60/100(60.0%)	42/100(42.0%)	0.006	71/100(71.0%)	60/100(60.0%)	0.001	71/100(71.0%)	42/100(42.0%)	<0.001
Specificity	49/61(80.3%)	51/61(88.5%)	0.227	46/61(75.4%)	49/61(80.3%)	0.250	46/61(75.4%)	51/61(88.5%)	0.008
PPV	60/72(83.3%)	42/49(85.7%)		71/86(82.6%)	60/72(83.3%)		71/86(82.6%)	42/49(85.7%)	
NPV	49/89(55.1%)	54/112(48.2%)		46/75(61.3%)	49/89(55.1%)		46/75(61.3%)	54/112(48.2%)	
Accuracy	109/161(67.7%)	93/161(59.6%)	0.132	117/161(72.7%)	109/161(67.7%)	0.330	117/161(72.7%)	93/161(59.6%)	0.013

*PPV* Positive predictive value, *NPV* Negative predictive value

**Fig. 3:**
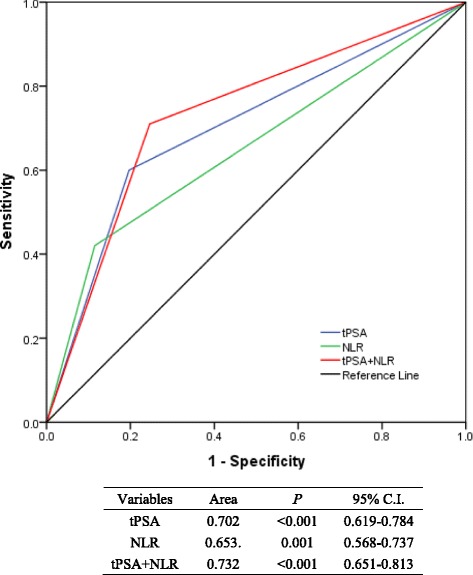
AUC model analysis of three methods (The blue, green and red lines stand for tPSA, NLR and tPSA + NLR respectively)

Given that AUC model analyses revealed that tPSA + NLR got the largest area of 0.732 (95% C.I. = 0.651-0.813, *P* < 0.001) in comparison with 0.702 (95% C.I. = 0.619-0.784, *P* < 0.001) for tPSA alone and 0.653 (95% C.I. = 0.568-0.737, *P* = 0.001) for NLR alone (Fig. 3).

## Discussion

Active surveillance for low risk localized prostate cancer with GS ≤ 6, has been verified to be a safe approach by several studies and may decrease its rate of overtreatment just basing on serum PSA and biopsy. The large prospective cohort study including 993 men conducted by Klotz L et al. [11] demonstrated that the safe period of time of AS had been prolonged to 15-year time; Another large cohort study including 731 men with low risk PCa who were randomly assigned to radical prostatectomy or intermediate follow-up revealed that there was no significantly reduction of all-cause or prostate-cancer mortality [12]. Screening potential patients with GS ≤ 6 for AS commonly depends on evaluation given by biopsy. However, the GS from prostate biopsy has an inherent sampling error and often differs from the GS in the prostate after radical prostatectomy (RP) [13]. According to Cookson MS et al., the biopsy GS differs as much as 60% to 70% from the GS in the RP specimen [14]. Another study conducted by Auvinen A et al. demonstrated that up to 20% of men with prostate cancer may be misdiagnosed at their first biopsy [15]. On the contrary, the accuracy of biopsy in comparison with postoperative histopathologic results in our study was only 46/161 (28.6%) with 108/161 (67.1%) underestimation and 7/161 (4.3%) overestimation respectively. The probable reasons were each core of biopsy took limited specimen which could only show the GS localized instead of that of whole lesions, and the location of tumor was unusual which was just outside of the biopsy grid [16]. Therefore, in order to increase the accuracy rate of biopsy, there is a need to find more applicable and available ways.

In addition, tPSA could not only be a potential predictor, demonstrated by logistic regression model, for discriminating real GS ≥ 7 Pca from those biopsy-based GS ≤ 6 PCa, but also could correctly identified 60% (60/100) GS ≥ 7 PCa and 80.3% (49/61) GS ≤ 6 PCa under cutoff value of 14.09 ng/ml in this study. Inflammation has been considered as a key contributor to carcinogenesis [17, 18]. Mounts of inflammatory cells, especially the neutrophil cell, infiltrated into the lesion had been verified by histopathology. Under stimulation of inflammation response executed by inflammatory cells and their releasing cytokines, cancer cells also generated several kinds of proinflammatory agents, such as IL-6, 12, TGF-β, TNF-α, CXC and CC, which could induced much more infiltration of inflammatory cells into tumor tissues [19]. A study conducted by Kwon YS et al. [20] demonstrated that instead of NLR, the baseline neutrophil cell and lymphocyte cell were superior predictors of biochemical recurrence, upstaging and associated with increased risk of men with prostate cancer under AS. According to Gokce MI [10], NLR is a predictor of GS upgrading and biochemical recurrence, but not disease upstaging in patients with low-risk PCa. However, neutrophil and lymphocyte counts were not found to be associated with GS upgrading, upstaging and biochemical recurrence, which was just opposite to Kwon YS. Besides, Thoma C [21] found that significant up-regulated PD-L1 on PCa is common and indicates poor prognosis. PD-L1 on tumor tissues is generated by exposure to IFN-γ, a typical proinflammatory agent, released by T cells. After combining with its ligand PD-1, the cytotoxic T cells were mediated to accelerated lysis and finally resulted in immune escape of cancer cells. Moreover, highly PD-L1 is also strongly associated with Enzalutamide resistant PCa [22, 23]. NLR, as a systemic inflammatory marker, has drawn much attention owing to its advantages of available, applicable with convenience and low cost. Elevated NLR strongly indicates poor outcomes of malignancy, and it could also serve as a biomarker to predict and discriminate the true nature of masses, especially in urinary, digestive and respiratory systems [24]. Gokce MI et al. [9] reported that NLR was found to differ with regard to histology of prostate biopsy and higher GS was associated with higher NLR in men with PCa, in which GS 8-10 group had a significantly higher mean NLR compared to GS 5-6 and GS 7 patients. Another large cohort conducted by Sonpavde Guru et al. [25] revealed that high NLR may be associated with an independent poor prognostic impact in post-docetaxel patients with mCRPC; Gazel E et al. demonstrated that NLR has the potential to be one of the parameters used in order to predict biochemical recurrence after radical prostatectomy with advantages of high sensitivity and specificity values [26]. All these studies above revealed that NLR might be a potentially useful marker for men with PCa in predicting and differentiating. In our study, higher NLR was significantly associated with GS ≥ 7 PCa in comparison with GS ≤ 6 PCa and it also could be a potential predictor for discriminating GS ≥ 7 PCa from biopsy-based GS ≤ 6 PCa.

Moreover, we extended the clinical usefulness of tPSA and NLR in form of their combination. Although combination of tPSA and NLR got the highest accuracy, it had no statistical significance in comparison with tPSA alone. However, this combination significantly increased the sensitivity of detecting GS ≥ 7 PCa in comparison with tPSA (*p* = 0.001) and NLR (*p* < 0.001) alone, and this results accorded with AUC model analyses in which combination of tPSA and NLR acquired the best diagnostic power with area of 0.732.

The main drawback of this study is its retrospective nature. Single center and design defects led the applicability of our conclusions remains restricted. The specific limitations follow as: 1. NLR is changeable according to the general conditions of patients, although we have ruled out apparent inflammation, hematological diseases, no NHT and other known conditions that will definitely influence the NLR, the specificity of NLR would also be affected by smoking behavior and even stress [27]; 2. As smoking history and alcohol history recorded in database were influenced by patients’ subjectivity, there was no doubt that the problems of self-report are inherent, such as lacking introspective ability to review relevant history accurately, responding by using their own rating scales, et al. All of them would lead response bias for the final results. 3. As fPSA was intact and not even tested for all 161 patients during primary physical examinations, f/t ratio included, it would lead the conclusion incomprehensive; 4. When we screened the enrolled patients, serum PSA and the numbers of positive cores were not restricted. As the cutoff value of tPSA over 10 ng/ml is more regional for practical use and current AS selection criteria was eligible for biopsy-based GS ≤ 6 and required tPSA < 10 ng/ml with positiv cores less or equal to 2 of 12 cores. Whether those uncontrolled parameters would influence our results or not remains unknown; 5. All patients came from a single institution. Therefore, prospective, multicenter trial and more stratification models are necessary to validate our findings.

## Conclusion

In conclusion, our study demonstrated that both tPSA and NLR might be potential predictors for discriminating real GS ≥ 7 Pca from those biopsy-based GS ≤ 6 PCa. Under the cutoff value, the sensitivity of tPSA for detecting patients with GS ≥ 7 from biopsy-based GS ≤ 6 PCa was much higher than that of NLR but lower than that of combination tPSA and NLR significantly. Besides, combination got the b provide us a new potential perspective to screen biopsy-based GS ≤ 6 PCa in order to make GS evaluation more accurate and decrease the mistake diagnostic rate in our clinical work. More stratification models and prospective studies are necessary.

## Abbreviations

AS: Active surveillance
GS: Gleason score
NHT: Neoadjuvant hormonal therapy
NLR: Neutrophil-lymphocyte ratio
PCa: Prostate cancer
PSA: Prostate-specific antigen
RP: Radical prostatectomy
TPSA: Total prostate-specific antigen
